# Advancing Gender Equity in International Eyecare: A Roadmap in Creating the Women Leaders in Eye Health (WLEH) Initiative

**DOI:** 10.3390/healthcare13131630

**Published:** 2025-07-07

**Authors:** Clare Szalay Timbo, Armaan Jaffer, Maria Jose Montero Romero, Gabriela Cubias, Heidi Chase, Sara T. Wester, Femida Kherani, Erin M. Shriver

**Affiliations:** 1 Orbis Canada, Toronto, ON M5T 2C2, Canada; 2 Faculty of Health Sciences, Queen’s University, Kingston, ON K7L 3N6, Canada; 3 Orbis International, New York, NY 10017, USA; 4 Seva Foundation, Berkeley, CA 94710, USA; 5 Bascom Palmer Eye Institute, University of Miami, Miami, FL 33136, USA; 6 Department of Ophthalmology and Visual Sciences, Faculty of Medicine, University of British Columbia, Vancouver, BC V5Z 3N9, Canada; 7 Department of Ophthalmology and Visual Sciences, Carver College of Medicine, University of Iowa, Iowa City, IA 52242, USA

**Keywords:** gender inequality, women in ophthalmology, leadership, virtual platforms, mentorship, global eye health, gender equity in eye health

## Abstract

Gender inequality remains a persistent issue in healthcare, especially in ophthalmology, where women face systemic barriers such as pay gaps, limited surgical opportunities, harassment, and unequal family expectations. Despite increasing entry into the field, women remain underrepresented in leadership, affecting career advancement and patient care. This study examines how virtual platforms, and co-led initiatives can address gender disparities in eye health. In 2021, Women in Ophthalmology, Seva Foundation, and Orbis International launched the Women’s Leaders in Eye Health (WLEH) initiative—a global community promoting mentorship, networking, and leadership development. Starting with virtual webinars and informal networking, the initiative expanded to in-person events in 2023 due to strong global engagement and demand. Early virtual programming, including webinars and “Coffee Hour” sessions, proved effective and laid the groundwork for broader offerings such as mentorship and professional development grants. WLEH’s success underscores the power of collaboration in promoting gender equity. By fostering connections and leadership pathways, WLEH offers a scalable model to break down gender challenges and uplift the next generation of women leaders to deliver more accessible eyecare globally.

## 1. Introduction

Gender inequality remains a pervasive issue in many fields, including healthcare, where systemic biases often hinder the advancement and well-being of women professionals. Cultural gender bias (i.e., accepted leadership styles for different genders, patriarchal norms, etc.) [1,2] and practical difficulties [3] in the workplace may significantly impact women eye healthcare professionals by isolating them and framing their challenges as individual problems [4]. This isolation can lead to burnout, reduced motivation, and dissatisfaction in a field where they would otherwise excel and find fulfillment [5]. Research highlights the significant underrepresentation of women in leadership roles within ophthalmology [6], which affects women’s career progression and overall hinders their advancement in the field. A recent study reveals that while men dominate executive roles in Canadian ophthalmology societies, both genders show similar research output, indicating a need to address ongoing gender disparities in leadership positions [7]. Additionally, a lack of diversity in decision-making spaces negatively affects not only health professionals and staff but also patients [8]. Ensuring gender equity means promoting women into leadership roles alongside their male peers and implementing policies that support the entire workforce in delivering high-quality care [9]. Gender-transformative change often requires a catalyst—such as a nonprofit organization or strategic leaders in the eyecare sector—that can challenge norms, mobilize resources, and inspire systemic shifts. On a global scale, forming a consortium can create a powerful movement to address gender inequity and counter common administrative resistance.

Today’s technology allows for the creation of a widely accessible virtual platform and dedicated space to focus on discussing gender issues, challenges, and solutions that can foster inclusivity and bring critical voices together to develop effective, strategic solutions. Fostering virtual platforms and connections can facilitate networking and mentorship, and thus help support women rise in leadership roles and provide opportunities for ongoing collaboration [10]. Additionally, virtual platforms and collaborative spaces provide a forum for sharing innovative strategies and best practices to overcome regional and global challenges in eyecare [11]. A digital platform can also promote equitable networking and research opportunities by overcoming gender disparities in access, funding and publication [3]. By uniting women across borders, such an initiative can tailor solutions to specific regional and global needs, ultimately driving progress and equity in eye health [10]. This collaborative approach accelerates advancement in the field and women’s roles within it.

In 2021, Women in Ophthalmology, the Seva Foundation, and Orbis International collaboratively launched a global, virtual initiative called “Women’s Leaders in Eye Health” (WLEH) [12]. This effort aimed to promote solidarity and support among female leaders in the eye health sector. While sessions initially began with formal webinars and informal “Coffee Hour” networking events, in 2023–2024, in-person events were piloted successfully. The creation of an international initiative for women in eye health has been well received by the high volume of initial ophthalmic participants in events, which speaks to the need to proactively address gender disparities to enhance global eyecare. WLEH is now determined to expand its programmatic offerings in response to the enthusiastic demand it has received and ensure more meaningful engagement across all ophthalmic cadres. The strong engagement from participants highlights the need for more diverse opportunities, and WLEH plans to introduce additional initiatives aimed at enhancing professional development, leadership training, and mentorship for women in global eye health. This expansion is aligned with the initiative’s mission to uplift women leaders and promote inclusive eyecare access for all.

This paper discusses the process through which WLEH was formed and provides insights from key programming initiatives in the four years since it was established. Sharing the WLEH model and lessons learned can further inspire female engagement in international eyecare leadership, enhance existing initiatives already working towards gender equity, and motivate new advocacy-oriented partnerships across global healthcare.

## 2. Creating the Women Leaders in Eye Health Initiative

**Figure 1:**
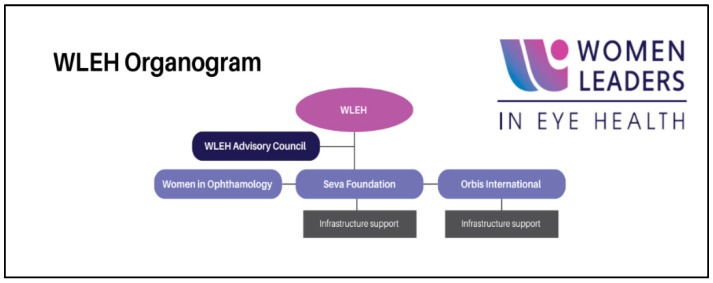
Organogram of the staffing and resource infrastructure of the WLEH initiative.

During the COVID-19 pandemic, discussions around supporting female eye health professionals globally were launched by Orbis International, a global international nonprofit that builds strong and sustainable eyecare systems globally, and Women in Ophthalmology (WIO), a powerful collective of women ophthalmologists, alongside Seva Foundation, a global nonprofit working to transform lives by restoring sight. Several advocates and gender champions within each of these organizations sought ways to collectively partner and utilize their platforms to uplift international colleagues and networks. Given the global pandemic, efforts focused on virtual connections and ways to practically support female eyecare professionals. In this collaborative effort between three non-profit organizations, WLEH was created through the launch of the first webinar series centered on gender equity in international eyecare and leadership. The series with 32 speakers (30 female, 2 male) received positive feedback, with 534 total attendees from 161 countries. Catapulting on the momentum of this series, the WLEH leadership team—composed of at least one member from each of the three non-profit organizations—led several similar in-person and virtual programs over the next two years. Recognizing the importance of continuing this work, all three non-profit organizations signed a formal Memorandum of Understanding in 2024, reaffirming the shared desire to advance the WLEH initiative further through innovative, inclusive, and engaging programming (Figure 1). In addition to centralizing back-end resources, fortifying administrative capacity, and formalizing the collaborative efforts, four main objectives were ratified in this agreement and a Monitoring and Evaluation (M and E) framework was developed in 2025 to measure impact and success. An advisory council of seven influential technical experts in the public health sphere launched at the end of 2024. This group provides strategic support to the initiative.

## 3. Funding

**Figure 2:**
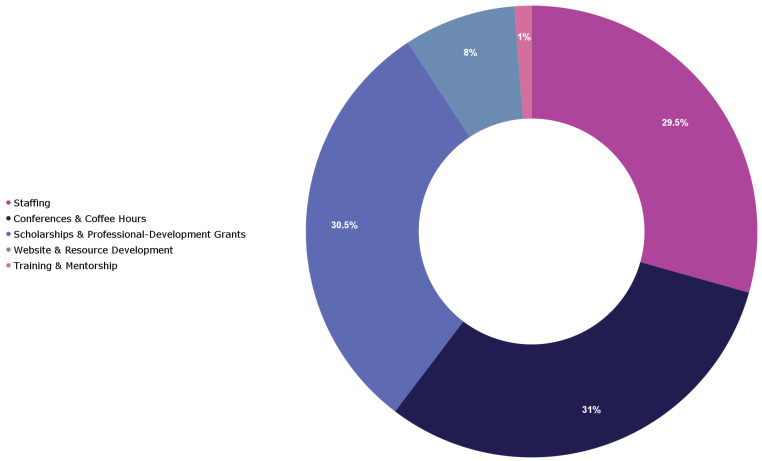
Allocation of WLEH’s operating budget, 2024.

In the first three years of the WLEH initiative, team members—including Orbis International and Seva staff, and a former WIO board member and WIO International Committee Chair—juggled their WLEH responsibilities alongside their other duties to launch the initiative. Programming was initially conducted virtually to reduce costs and adhere to the travel restrictions and global lockdowns imposed by COVID-19. WLEH secured formal funding through a two-year operating grant for 2024–2025. During the first grant year, the initiative utilized 31% of total funds on core program delivery (Figure 2). Approximately 70.5% of 2024’s expenditure was directed to programmatic activities—conferences, Coffee Hour sessions, professional-development grants, and training and mentorship. The remaining funds are reserved for 2025 to expand geographic reach, reinforce existing programs, and initiate additional gender-equity efforts. This initial grant enabled the launch of WLEH Professional Development grants and the expansion of in-person Coffee Hour events at several international conferences. All three partner organizations continue to contribute substantial in-kind staff and committee time; however, Orbis International and the Seva Foundation now provide enhanced institutional support for logistics, coordination, and financial tracking (Figure 1, “infrastructure support”).

## 4. WLEH’s Four Main Objectives

The following sections outline the activities and approaches for programming related to WLEH’s four main objectives (Table 1) and describe, for each objective, the corresponding indicators that will be tracked under the M and E framework.

### 4.1. Objective 1: Conference Support, Participation, and Thought Leadership

In early 2024, the WLEH team initiated a plan to establish professional development grants for female eye health professionals who submitted abstracts, posters, or research for international or regional eye health conferences. During 2024, WLEH awarded grants to 15 recipients across four conferences. The application process involved anonymizing submissions, with one reviewer from each organization evaluating all applications using standardized rubrics for grading. Grants were awarded based on the highest cumulative scores and the applicants’ ability to travel, primarily contingent on visa approvals. For 2025, the process will be refined with advanced timelines to accommodate more applicants and align with conference abstract deadlines. Progress toward this objective will be assessed through the M and E framework by tracking the number of professional-development grants issued, the proportion of female authors and speakers at partner conferences, and year-on-year changes in women’s representation on conference panels.

### 4.2. Objective 2: Coffee Hours, Webinars, and Themes Resources

**Figure 3:**
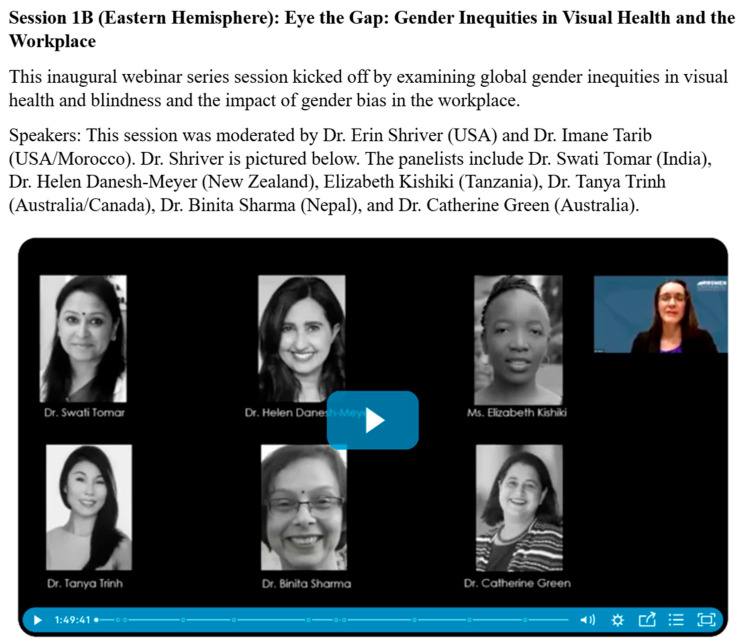
Screenshot of speakers and facilitator from the Session 1B of the original WLEH webinar, 2021.

Starting in 2021, the WLEH collaboration launched a four-part webinar series featuring 32 global panelists. The sessions focused on exploring global gender disparities in visual health and within the workplace and offered strategies for female eye health professionals to advance their careers. See Figure 3 and Table 2 below for more details on the initial virtual webinar series.

After the success of the initial webinar series in the fall of 2021, the WLEH team responded to the request for more sessions discussing similar topics. The webinar series evolved into the WLEH Coffee Hours. Coffee Hours are informal, synchronous, virtual or in-person sessions hosted by panelists speaking on a specific topic. From 2022 to 2023, a total of eight virtual Coffee Hours were hosted for global audiences. In addition, in both years, WLEH piloted livestreamed events from two locations as part of Orbis International’s Flying Eye Hospital programming in Doha Qatar (2022) and Lusaka, Zambia (2023). An initial in-person Coffee Hour event was piloted during the College of Ophthalmology of Eastern, Central and Southern Africa’s (COECSA) regional ophthalmology conference in Mombasa, Kenya, in August 2023. With additional resources and funding, in-person events expanded in 2024, and a total of six live-in-person Coffee Hours were hosted at international conferences. Speakers and facilitators for Coffee Hours were invited to contribute based on their areas of expertise and availability. Male speakers were engaged more formally for the in-person Coffee Hours in 2024 intentionally, and key male allies were invited to participate in the discussions. Additionally, in 2024, four WLEH Professional Development grantees had the opportunity to speak at in-person Coffee Hours, providing a platform to showcase their leadership and share their unique perspectives and expertise. Across all WLEH platforms, we intentionally curate speakers who span regions, resource settings, professions, ages, and ethnic backgrounds, ensuring that a broad spectrum of intersecting challenges and lived experiences faced by women in eye health is recognized and amplified. The M and E framework will measure success via counts of Coffee Hour and webinar sessions, participant diversity and attendance rates, and post-event feedback scores that evaluate knowledge gain and networking value. See Table 3 below for more details on the Coffee Hours.

### 4.3. Objective 3: Establishing Ophthalmic Representation on Key Global Gender Organizations

In 2024, the WLEH initiative’s three partner organizations united to nominate international leaders for the formation of an initial “Advisory Council” (Figure 1). Leaders from diverse sectors and regions were considered, and seven initial individuals were selected to help establish this group. Recognizing the intersectoral challenges that females face globally, a diverse body was intentionally selected. The goal of the WLEH Advisory Council is to provide strategic representation that will build awareness and momentum for the initiative within influential and decision-making spaces. Council members hold a range of positions and connections, offering valuable linkages and networking opportunities for the broader WLEH community. Ideally, the advisors will also facilitate sponsorships and nominations for key leadership roles such as committee members, board members, and executive team leaders in various spaces. The nascent Advisory Council was established by early 2025 and is actively offering strategic support and guidance to advance the initiative. Indicators for this objective include Advisory Council membership diversity, the number of external leadership nominations facilitated, and the percentage of council recommendations adopted by partner bodies.

### 4.4. Objective 4: Building Volunteer Faculty Pool, Training, and Gender Resources

Building upon the expanded programming in 2024, this initiative seeks to establish a global directory of female eye health professionals who can provide technical clinical training along with support around leadership, mentorship, the formation of female-led societies and organizations, gender equity, and beyond. The team leading this effort is also collaborating with global partners to better understand and address requests for support related to localized or cultural gender inequities, ensuring that female eye health professionals receive the specific help needed to overcome challenges in these contexts. Additionally, the team is working on translating into English and creating an online training course that highlights the links between eye health conditions, treatments, and gender inequities worldwide. This course, already available in Spanish via Cybersight, has been well-received in Latin America for its practical approach and focus on fostering gender-transformative change in eyecare, aiming to further support and educate professionals on these critical issues. The M and E framework will track faculty-pool growth and regional balance, completion of gender-transformative training modules, and participant self-reported gains in leadership competence following each online training course.

## 5. Lessons Learned and Next Steps to Further Empower Female Leaders

With just over four years of experience and the WLEH programming accomplished thus far, there are some valuable lessons that have been learned and next steps to share in how to best uplift female eye health leaders gleaned from the events and sessions.

Four critical lessons learned can be gleaned from the initial launch of the WLEH initiative:

1. **Virtual Platforms Are Accessible for Engagement**: The success of the initial virtual webinars and Coffee Hours underscores the importance of digital platforms in fostering global connections. These platforms allowed for cost-effective engagement and facilitated networking and mentorship across borders despite limitations imposed by COVID-19 [10]. Continuing to use virtual platforms allows for accessibility and inclusivity to foster global connections and networking for women eye health professionals. However, persistent challenges are the limited accessibility of reliable internet and the complexity of coordinating across time zones, which can hinder consistent participation in virtual meetings for a global audience.
2. **Diversity in Leadership Enhances Impact**: The creation of the WLEH initiative demonstrated that involving diverse leaders enhanced the initiative in the same way that this initiative advocates for diversity in leadership. This approach helps address gender disparities and promotes more inclusive and effective programming [8]. Given the current pushback against Diversity, Equity and Inclusion initiatives as of 2025, prioritizing inclusivity and diversity remains a critical—yet often under-supported—effort in many spaces.
3. **Strategically using Funds to Demonstrate Growth**: Early on, WLEH strategically used resources to demonstrate the potential of this initiative and leveraged outcomes by finding additional funding support. The shift from virtual to in-person events and the introduction of new programs like the WLEH Professional Development grants has highlighted strategic growth and impact. Thus, allocating other resources and funding is essential for expanding the initiative’s reach and impact, allowing for the development of new programming and support mechanisms [5]. However, finding and securing funding resources remains a relevant challenge given the cuts made by governments globally in relation to funding for the wider development sector.
4. **In-Person Events Foster Stronger Connections**: The transition from virtual to in-person events revealed the value of face-to-face interactions. Live sessions and conferences provided invaluable networking opportunities and allowed for deeper engagement with participants, reinforcing the importance of creating spaces where women in eye health can connect directly and share experiences [11]. Challenges persist in fostering and sustaining meaningful connections in a timely and effective way, given geographical, and scheduling differences faced by a global audience of busy health professionals.

Moving forward, there remains a need for further infrastructure changes within the wider eye health sector. With continued support, funding, and a strategic approach, WLEH is set to transform the landscape of eye health leadership, ensuring that women’s contributions are recognized and valued globally. Formal leadership roles must be assigned to women, and they need support to thrive in these challenging positions [9]. To address these issues, WLEH must strengthen its networks with formal institutions to advocate for policy changes and improvements based on the feedback shared during Coffee Hours and in other forums. While preliminary indicators—including strong global engagement, overwhelming interest in professional-development grants, a growing roster of partner collaborations, and renewed funding commitments—suggest early enthusiasm, this commentary is intentionally descriptive and does not constitute a formal qualitative or quantitative evaluation of WLEH’s impact. Internally, program success will be assessed through a robust M and E framework. Further, as program delivery progresses, participant-level outcomes will be examined in forthcoming mixed-methods studies, which will similarly be shared in peer-reviewed academic journals.

## 6. Conclusions

The WLEH initiative demonstrates a successful model for advancing gender equity and leadership in eyecare so that other organizations can adapt to their local and global contexts. By leveraging virtual platforms, enhancing advocacy efforts, and expanding support networks, WLEH provides a framework for creating impactful opportunities for female eye health professionals. To increase visibility and attract potential partners, strategic advertising and targeted outreach campaigns can also be considered as part of this model. We encourage health professionals, organizations, and stakeholders to consider partnering and implementing similar strategies to foster an inclusive and equitable environment in other sectors. This approach will help inspire and support the next generation of leaders, driving systemic change and elevating women’s contributions on a global scale.

## Figures and Tables

**Table 1:** WLEH objectives, 2024.

No.	Theme	Goal
**1**	Conference Support, Participation and Thought Leadership	To provide female eye health professionals with opportunities to attend, present, and share their original research at conferences, thereby facilitating strategic networking and career development.
**2**	Coffee Hours, Webinars and Themes Resources	To create supportive spaces where female eye health professionals can share their experiences and advice, fostering meaningful connections and authentic relationships that promote career growth and mutual support.
**3**	Establishing Ophthalmic Representation on Key Global Gender Organizations	To establish a council that enhances sector-wide connections and linkages, strategically positioning female eye health leaders for nomination and representation in decision-making roles and authoritative positions.
**4**	Building Volunteer Faculty Pool, Training, and Gender Resources	To increase the recruitment of female eye health professionals for clinical and leadership roles and to develop and disseminate training materials on gender in eye health for global learners.

**Table 2:** WLEH Live Synchronous Webinars with Panels in 2021.

Event Title and Target Audience	Attendance	Countries
Eyeing the Gap: Gender Inequities in Visual Health and the Workplace—Session 1A (Western Hemisphere)	197	52 countries
Eyeing the Gap: Gender Inequities in Visual Health and the Workplace—Session 1B (Eastern Hemisphere)	101	35 countries
Catapulting Your Career: Seeking Out Opportunities for Growth—Session 2A (Western Hemisphere)	160	46 countries
Catapulting Your Career: Seeking Out Opportunities for Growth—Session 2B (Eastern Hemisphere)	76	28 countries

**Table 3:** WLEH Coffee Hours (2022–2024) details.

Date	Session Topic	Format	Location
**February 2022**	Oculoplastic	Virtual	
**April 2022**	Glaucoma	Virtual	
**June 2022**	Cataract	Virtual	
**September 2022**	Mentorship	Virtual	
**November 2022**	Simulation Training	Streamed live	Doha, Qatar
**February 2023**	Leadership (Eastern Hemisphere)	Virtual	
**February 2023**	Leadership (Western Hemisphere)	Virtual	
**May 2023**	Diversity, Equity and Inclusion (Eastern Hemisphere)	Virtual	
**May 2023**	Diversity, Equity and Inclusion (Western Hemisphere)	Virtual	
**August 2023**	Mentorship	In person	COECSA conference, Mombasa, Kenya
**September 2023**	Mentorship	Streamed live	Lusaka, Zambia
**April 2024**	Simulation Training	In person	ASCRS conference, USA
**June 2024**	Gender Equity with IAPB Gender Equity Workgroup	In person	IAPB conference, Mexico
**July 2024**	Sharing Solutions and Space	In person	AOC conference, Rwanda
**August 2024**	Considerations for Women in International Ophthalmology	In person	GOS conference, USA
**August 2024**	Global Gender Equity Connections and Strengthening Networks	In person	WOC conference, Canada
**October 2024**	Empowering Diverse Voices	In person	AAO conference, USA
**December 2024**	Alternative Career Choices	Virtual	

Acronyms: College of Ophthalmology of Eastern and Southern Africa Conference (COECSA), American Society of Cataract and Refractive Surgery (ASCRS), The International Agency for the Prevention of Blindness (IAPB), African Ophthalmology Council (AOC), Global Ophthalmology Summit (GOS), World Ophthalmology Council (WOC), and the American Academy of Ophthalmology (AAO).

## Data Availability

Not applicable.

## Abbreviations

The following abbreviations are used in this manuscript:

AAO	American Academy of Ophthalmology
AOC	African Ophthalmology Council
ASCRS	American Society of Cataract and Refractive Surgery
COECSA	College of Ophthalmology of Eastern and Southern Africa Conference
GOS	Global Ophthalmology Summit
IAPB	The International Agency for the Prevention of Blindness
M and E	Monitoring and Evaluation
WIO	Women in Ophthalmology
WLEH	Women Leaders in Eye Health
WOC	World Ophthalmology Council
